# Enhancing higher-order modal response in multifrequency atomic force microscopy with a coupled cantilever system

**DOI:** 10.3762/bjnano.15.57

**Published:** 2024-06-17

**Authors:** Wendong Sun, Jianqiang Qian, Yingzi Li, Yanan Chen, Zhipeng Dou, Rui Lin, Peng Cheng, Xiaodong Gao, Quan Yuan, Yifan Hu

**Affiliations:** 1 School of Physics, Beihang University, Beijing 100191, P.R. China; 2 State Key Laboratory of Catalysis, CAS Center for Excellence in Nanoscience, Dalian Institute of Chemical Physics, Chinese Academy of Sciences, Dalian 116023, P. R. China

**Keywords:** atomic force microscopy, coupled system, higher-order modes, macroscale, multifrequency AFM

## Abstract

Multifrequency atomic force microscopy (AFM) utilizes the multimode operation of cantilevers to achieve rapid high-resolution imaging and extract multiple properties. However, the higher-order modal response of traditional rectangular cantilever is weaker in air, which affects the sensitivity of multifrequency AFM detection. To address this issue, we previously proposed a bridge/cantilever coupled system model to enhance the higher-order modal response of the cantilever. This model is simpler and less costly than other enhancement methods, making it easier to be widely used. However, previous studies were limited to theoretical analysis and preliminary simulations regarding ideal conditions. In this paper, we undertake a more comprehensive investigation of the coupled system, taking into account the influence of probe and excitation surface sizes on the modal response. To facilitate the exploration of the effectiveness and optimal conditions for the coupled system in practical applications, a macroscale experimental platform is established. By conducting finite element analysis and experiments, we compare the performance of the coupled system with that of traditional cantilevers and quantify the enhancement in higher-order modal response. Also, the optimal conditions for the enhancement of macroscale cantilever modal response are explored. Additionally, we also supplement the characteristics of this model, including increasing the modal frequency of the original cantilever and generating additional resonance peaks, demonstrating the significant potential of the coupled system in various fields of AFM.

## Introduction

Multifrequency atomic force microscopy (AFM) has become an important tool for nanoscale imaging and characterization [1–2]. This technique involves the excitation and detection of multiple frequencies to improve data acquisition speed, sensitivity, and resolution, as well as to enable material properties mapping with additional contrast [3]. Among the various multifrequency AFM methods, bimodal AFM is the most commonly used, which relies on the excitation and detection of two resonant frequencies [4]. Most studies have focused on the first two eigenmodes of the cantilever, with the fundamental resonant mode tracking the sample topography and the higher-order resonant mode providing information about mechanical properties [5–6]. Bimodal AFM is relatively simple to operate and offers improved imaging quality. Hence, it is widely applicable in diverse fields such as physics, chemistry, and biology [7–9].

The higher-order eigenmodes of the cantilever can effectively improve Q-factor, imaging rate, and mass sensing resolution [10–11]. For traditional rectangular cantilevers, the higher-order modal response is usually weaker than that of the fundamental mode in ambient air [12]. This reduction negatively impacts detection sensitivity and limits the application of multifrequency AFM. To promote the application of multifrequency techniques, researchers have proposed various methods to improve the higher-order mode response by modifying cantilever beams. For example, researchers have achieved this by etching specific regions of the cantilever, coating, and utilizing magnetostrictive actuation to enhance the resonance modes of individual cantilevers [13]. Some have explored the enhancement of modal properties by adding rebar structures to cantilever beams using 3D laser writing [14]. Moreover, the V-shaped design of the cantilever beam reduces its frequency ratio and enhances the possibility of self-excitation, making it more appropriate for multifrequency AFM, particularly in the realm of bimodal AFM [15]. In addition, there are other ways to enhance the resonance response by changing the cantilever mass distribution so that the higher-order modal frequencies are integer multiples of the fundamental frequency, such as adding an inner paddle [16], attaching finite size masses [17], and cutting a rectangular slot in the cantilever [18]. However, most of the above methods involve micromachining the cantilever, which is costly and complex.

To develop a simpler method to enhance the higher-order modal response of cantilever, we previously proposed a model based on a bridge/cantilever coupled system [19]. The model divides the rectangular cantilever into two parts, namely, the left clamped bridge part and the right simply supported cantilever part. By adjusting the left bridge part to align with a higher resonance mode of the right cantilever, the coupling effect enhances the higher-order modal response. Additionally, in the field of high-frequency fast imaging, most studies focus on increasing the modal frequency by decreasing the cantilever size to achieve faster scanning speeds [20–22]. According to our further study, the bridge/cantilever coupled system model can also significantly increase the modal frequency of the cantilever, providing a new idea for high-frequency fast imaging. The structure of this model is simple, eliminating the need for micromachining of the cantilever, resulting in low cost, and facilitating widespread utilization. However, previous studies have only focused on theoretical analysis and preliminary simulations under ideal conditions. The enhancement effect of higher-order modes has not been compared with traditional cantilever beams. Therefore, further simulation analyses and experiments are required to validate the practical application of this model.

In this work, all research is based on an enlarged macroscale cantilever, which was proportionally scaled up from a multifrequency AFM microscale cantilever. The reliability of the coupled system theory is demonstrated by exploring the macroscale modal response, which provides pre-validation for subsequent microscale applications. Therefore, we established a macroscale experimental platform to emulate the modal response of microscale cantilevers in real-world environments. Comprehensive and realistic simulation and experimentation were conducted to provide a more thorough investigation. Comparing the modal response capability with traditional cantilever beams confirmed the enhanced effect of higher-order modal response in the coupled system model. Also, an analysis of the influence of the length variations on both sides and the excitation positions on higher-order modal response was performed. Furthermore, the coupled system exhibited unique characteristics such as high modal frequency and additional resonance peaks, expanding its potential range of applications.

## Results and Discussion

### Transfer function analysis

**Figure 1:**
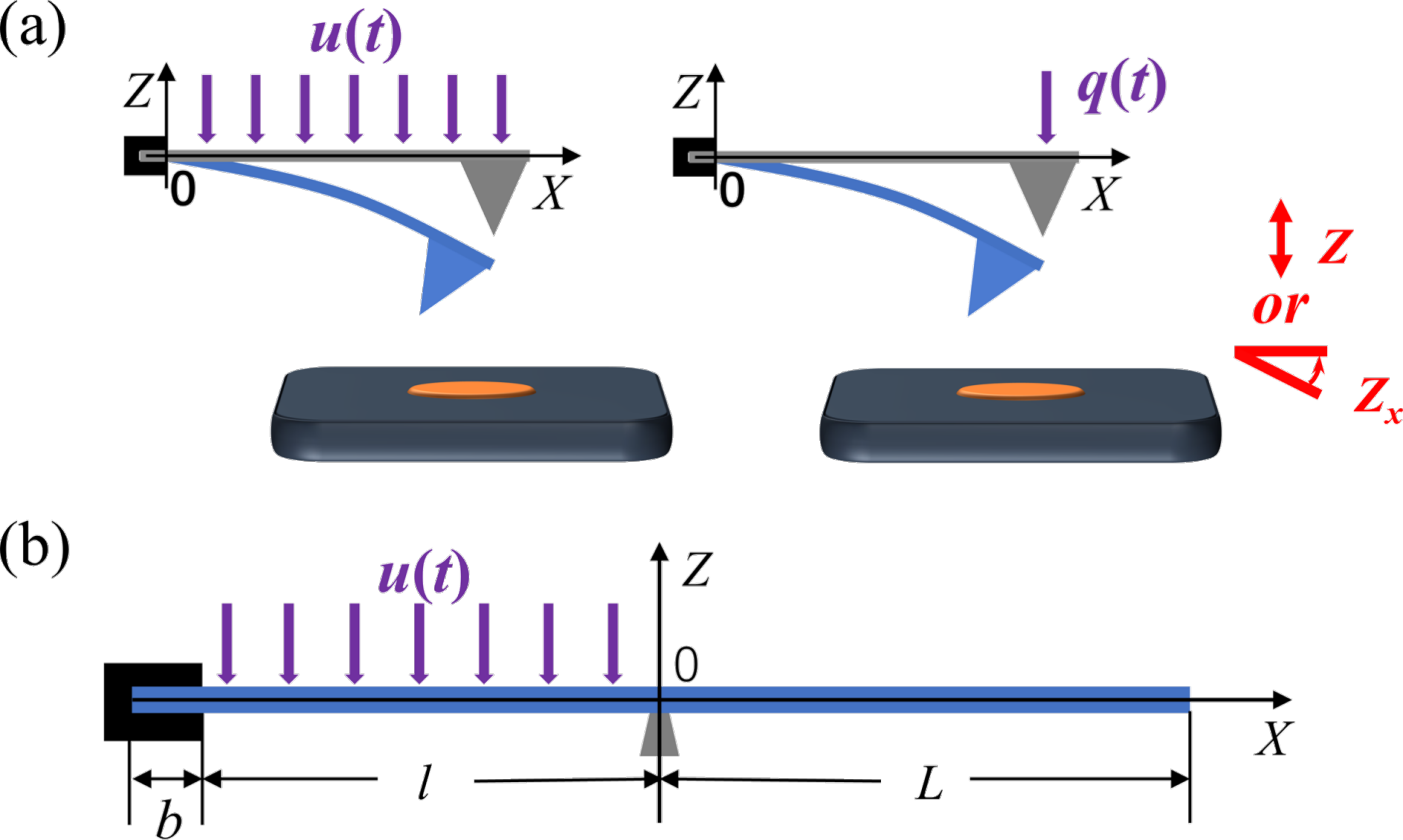
(a) Microcantilever beams with different inputs (purple) and outputs (red). (b) Diagram of the bridge/cantilever coupled system.

For a traditional rectangular cantilever beam, the dynamics of the system can be described by transfer functions [12,23]. Representing the cantilever as an isolated input/output system, the input quantities are the excitation force *u*(*t*), uniformly distributed along the cantilever beam, and the external point force *q*(*t*), acting on the tip of the cantilever. The output quantities are the vertical deflection *Z* and the slope of the deflection *Z_x_* at the free end of the cantilever beam. They are shown in Figure 1a. The Bode plots of the non-contact tip–sample interaction [12] and the contact tip–sample interaction [23] can be obtained through transfer function analysis. The results indicate that the higher-order modal response of the traditional rectangular cantilever gradually weakens.

Our previously proposed bridge/cantilever coupled system is shown in Figure 1b. This model divides the rectangular cantilever beam into two parts by applying a support to the bottom surface while applying an excitation force to the left part. With the bottom support point of the cantilever as the zero point, the lengths of the left and right sides are *l* and *L*, respectively. The cantilever has a width of *w*, a thickness of *d*, and a constrained length of *b* for clamping the probe. In order to simplify the model solution, we did not consider the external point force at the tip of the cantilever.

More details about the derivation process can be found in [19]. The final result for the transfer function *G* of the infinite product expansion is obtained as:


[1] $$\begin{array}{c} G=[\left(\frac{z^{3}}{3}\prod_{n=1}^{\infty }\left[1+\frac{z^{4}}{4d_{n}^{4}}\right]\right)\left(\left(2\prod_{n=1}^{\infty }\left[1+\frac{4z^{4}}{{\left(2n-1\right)}^{4}\pi^{4}}\right]\right)\left(2z\prod_{n=1}^{\infty }\left[1+\frac{z^{4}}{4b_{n}^{4}}\right]\right)\right)\\ -\frac{1}{2}\left(4z\prod_{n=1}^{\infty }\left[1+\frac{{\left(2z\right)}^{4}}{4b_{n}^{4}}]\right)\right]/2z\prod_{n=1}^{\infty }\left[1+\frac{z^{4}}{4b_{n}^{4}}\right], \end{array}$$

where


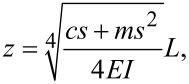


*s* is the (complex) Laplace variable, and *m* is the mass per unit length. *EI* and *c* are the flexural stiffness and the damping coefficient, respectively. *d_n_* and *b_n_* satisfy the following expressions:


[2] $$\tan d_{n}=\tanh d_{n},\;d_{n}>0,\text{ real,}$$

[3] $$\tan b_{n}=-\tanh b_{n},\;b_{n}>0,\text{ real.}$$

**Figure 2:**
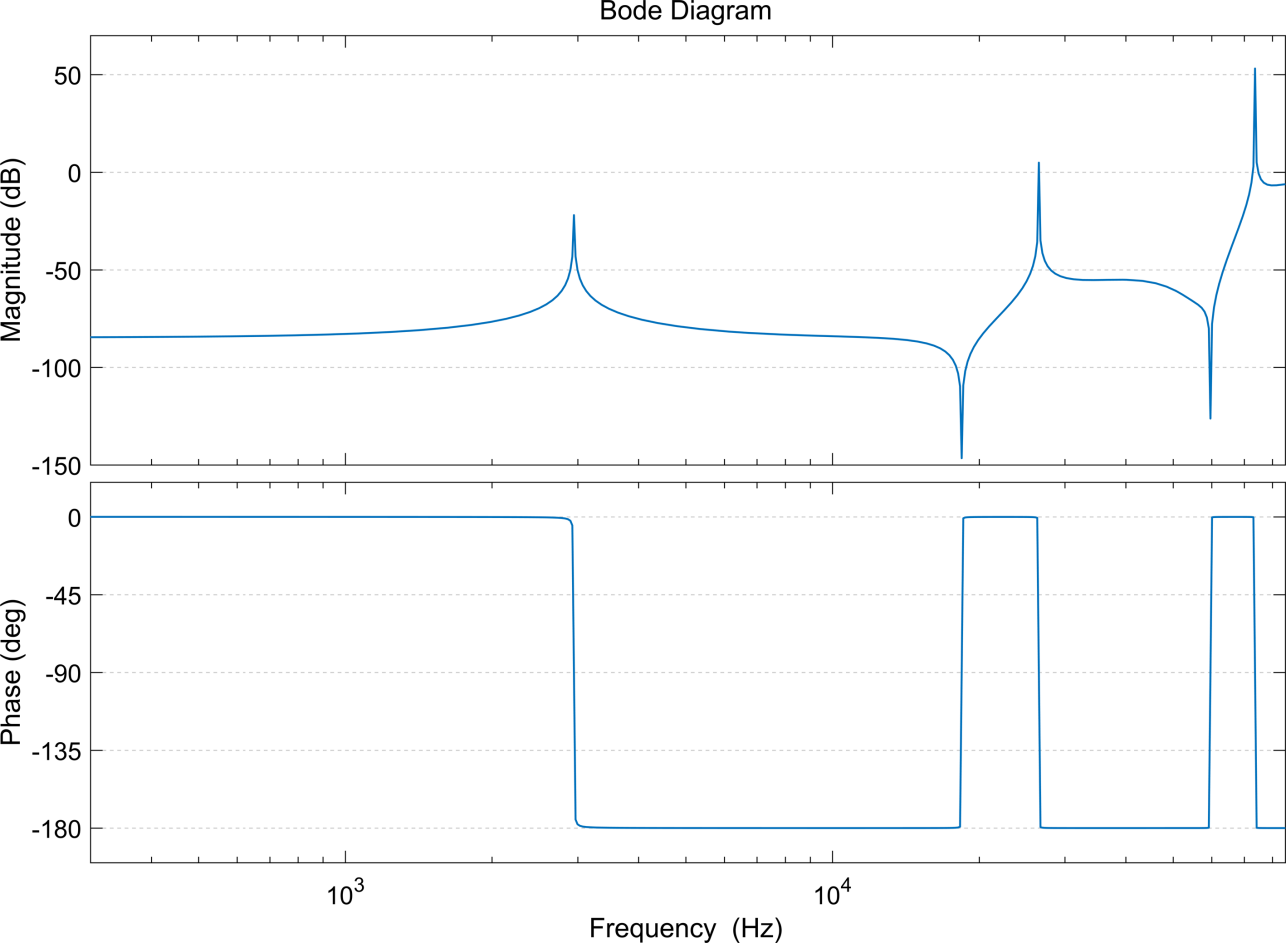
Bode plot of the response of the macroscale bridge/cantilever coupled system.

Assuming *l* = *L* and using the macroscale cantilever parameters studied in this paper, that is, *E* = 166 GPa, *m* = 0.04 kg/m, *L* = 2.75 cm, *w* = 1 cm, *d* = 1.8 mm, and *c* = 0.01 kg/ms, the Bode plot can be obtained as shown in Figure 2. The result shows that the higher-order modal response of the coupled system gradually increases, which will improve the sensitivity of the detection and promote the development of multifrequency AFM utilizing higher-order modes of the cantilever to image sample properties.

### Finite element analysis

#### Model size and simulation parameters

Several commercial rectangular multifrequency AFM cantilevers of different sizes from different manufacturers are summarized in [24]. In the ANSYS Workbench finite element simulation, the material used in the simulation is silicon, with a Young's modulus of 166 GPa and a density of 2330 kg/m^3^. We applied the bridge/cantilever coupled structure to microcantilevers with different sizes and found that the modal vibration shapes were all in accordance with the vibration characteristics of the coupled system; also, the modal frequencies and higher-order modal responses were enhanced. This indicates that the model has no special requirements for the cantilever size. The first two orders of the simulated modal vibration shapes for different sizes of microcantilevers are shown in Supporting Information File 1. Considering the need to scale up and experiment, we selected cantilevers of the same size from the manufacturers BudgetSensors (Tap190-G) and NanoWorld (NW-SSS-NCL) for our study. The selected cantilever dimensions are as follows: a length of 225 ± 10 μm, a width of 38 ± 5 μm, and a thickness of 7 ± 1 μm. The finite element analysis result shows that the original first and second modal frequencies of the cantilevers are 201.8 and 1259.3 kHz, respectively, and after applying the bridge/cantilever coupling system, the modal frequencies are 716.7 and 4356.3 kHz, respectively. The ratio between the higher-order modal frequency and the fundamental eigenfrequency decreases from 6.24 to 6.08, approaching an integer value. This reduction in frequency ratio enhances the high harmonic oscillations and improves the phase contrast during imaging [15].

**Figure 3:**
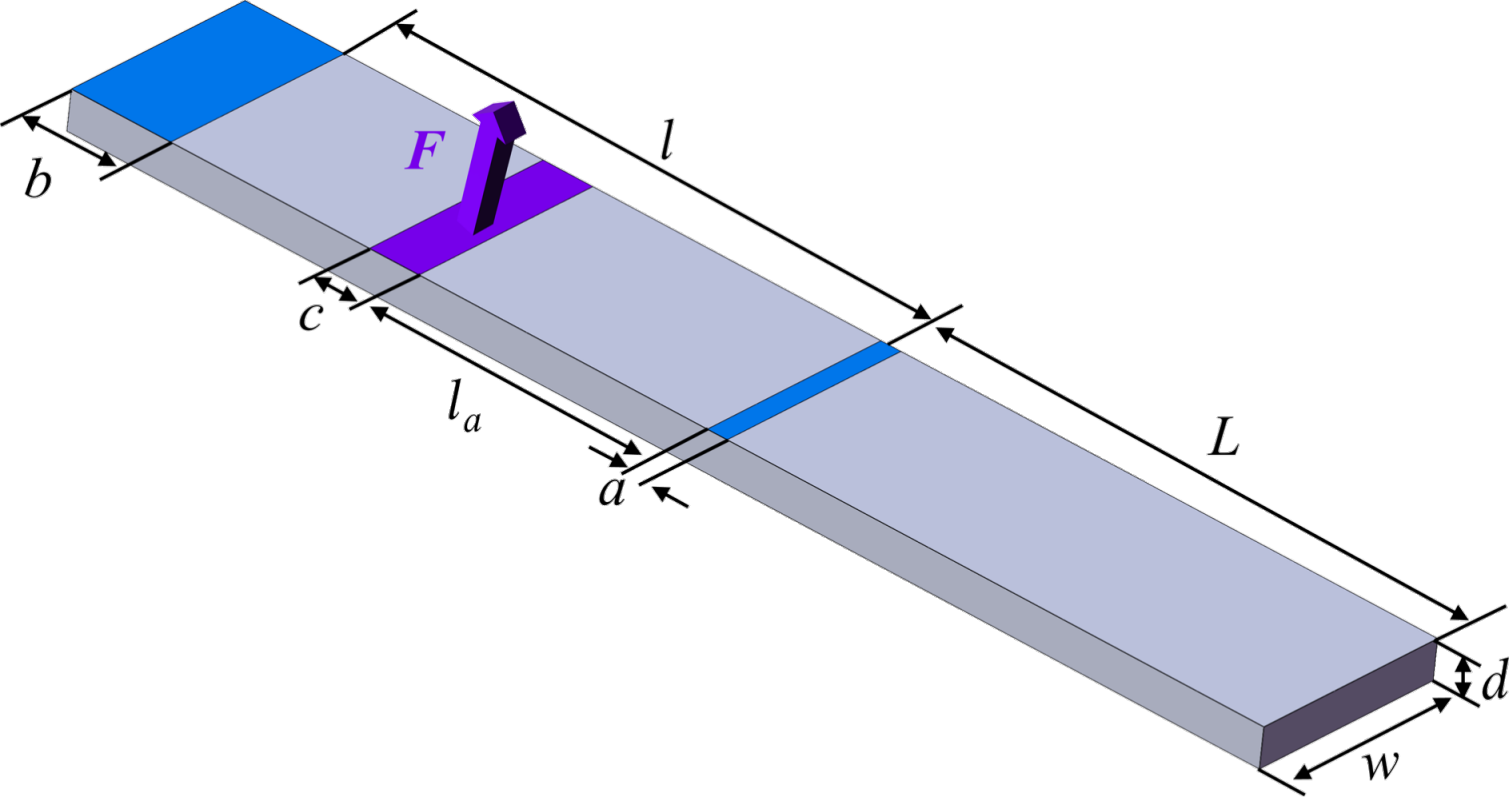
Lower surface schematic diagram of the macroscale bridge/cantilever coupled system model. The total length is 6 cm, with width *w* of 1 cm and thickness *d* of 0.18 cm. The location and size of constraints (blue) and excitations (purple) are labeled.

The macroscale cantilever model (6 × 1 × 0.18 cm) is 266 times the size (within the error range) of the microscale cantilever dimensions. Considering the effect of clamping the probe on the modal response of the cantilever in practice, we define a cantilever model with left clamping fixation and selective support in the center; its lower surface is shown in Figure 3. When the bottom surface is not constrained by the support, it is the traditional rectangular cantilever beam model. Assuming that the initial lengths of the left and right sides are equal, the parameters labeled in Figure 3 are shown in Table 1, where *a* and *b* (blue) are the dimensions of the bottom support and the fixed end of the cantilever, respectively, *l_a_* is the distance from the excitation position to the bottom support position, and *c* (purple) is the size of the excitation surface width. Table 1 lists three excitation sizes, using mostly 0.25 cm. In the simulation, the magnitude of the applied excitation force *F* is 1 N. In order to better show the trend of modal enhancement, we take the logarithm of the amplitude values of all the simulation results with a base of 10.

**Table 1:** Macroscale cantilever model parameters.

Parameter	Value [cm]

*L*	2.75
*l*	2.75
*w*	1.0
*d*	0.18
*a*	0.1
*b*	0.5
*c*	0.25/2.0/5.0

#### Modal response comparison

First, through modal analysis, we obtained the first- and second-mode frequencies of a traditional rectangular cantilever beam as *f*_1_ = 816.0 Hz and *f*_2_ = 5090.2 Hz, respectively. Similarly, for the bridge/cantilever coupled system, the first- and second-mode frequencies were determined as *f*_1_ = 2708.8 Hz and *f*_2_ = 16615.3 Hz, respectively. It is noteworthy that the modal frequency of the coupled system is more than three times higher, and the ratio between the first two modal frequencies decreases from 6.24 to 6.13.

**Figure 4:**
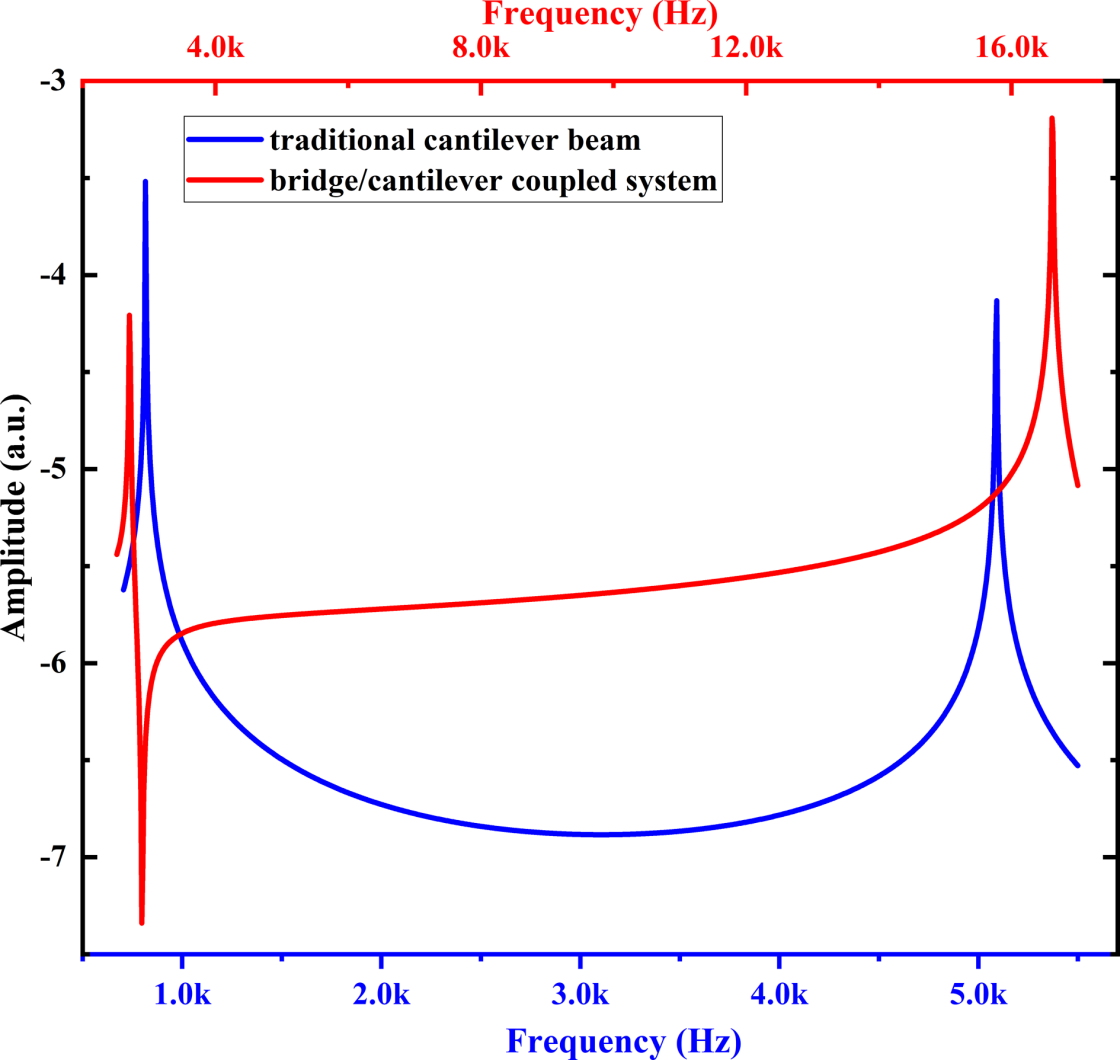
The first two orders of modal frequency response of a traditional rectangular cantilever beam (blue) and the coupled system (red).

Following this, the first two orders of modal responses were obtained through frequency response analysis, and the results are shown in Figure 4. For the traditional cantilever beam, the used excitation method was the most common whole face excitation, while for the coupled system, left face excitation was used. As illustrated in Figure 4, the modal response of the coupled system is gradually elevated. This observation is consistent with the results of transfer function analysis and contrary to the traditional cantilever beam modal response. Additionally, it is noteworthy that the second-order modal response amplitude of the coupled system (6.43 × 10^−4^ mm) is 8.72 times that of the traditional cantilever (7.37 × 10^−5^ mm). That is, the second-order modal response of the coupled system is improved by a factor of 7.72. It even exceeds the first-order modal response amplitude of the traditional cantilever (3.03 × 10^−4^ mm). The gap between the responses of the third- and higher-order modes is even bigger. These findings provide valuable insights into the different modal responses between the coupled system and the traditional cantilever.

Additionally, it can be observed from the graph that the first-mode response of the coupled system is reduced, indicating a certain limitation on the first-mode response in the coupled system. This phenomenon may be related to the excitation position. However, it does not have a large impact on imaging in practical applications because the first mode of the cantilever is used for topographic imaging with simpler information components.

#### Optimal conditions for modal response

To obtain the optimal conditions for the higher-order modal response of the bridge/cantilever coupled system, the left and right lengths and excitation positions were simulated and analyzed.

The first two orders of modal frequencies for different left side lengths *l* obtained through modal analysis are shown in Table 2. The total length of the cantilever is kept constant during the simulation, that is, the right side length *L* keeps changing with the left side length *l*. The results show that the modal frequency increases as the left side length *l* increases. When the left side length *l* and the right side length *L* are equal (*l* = *L* = 2.75 cm), the modal ratio is minimized, thereby providing a greater potential for self-excitation. Furthermore, in the simulation, we find that the coupled system generates an additional mode near the second mode. The modal frequency decreases with increasing *l*, and when *l* is 3 cm, its modal frequency (15884.3 Hz, not listed in Table 2) is already lower than that of the second mode (24178.6 Hz).

**Table 2:** Simulation results for frequencies and ratios of the first two modes with different lengths of the left side *l*.

*l* [cm]	*f*_1_ [Hz]	*f*_2_ [Hz]	modal ratio

1.50	1538.3	9570.6	6.22
2.00	1999.6	12408.2	6.21
2.50	2706.8	16598.5	6.13
2.75	3209.3	18077.3	5.63
3.00	3867.1	24178.6	6.25

**Figure 5:**
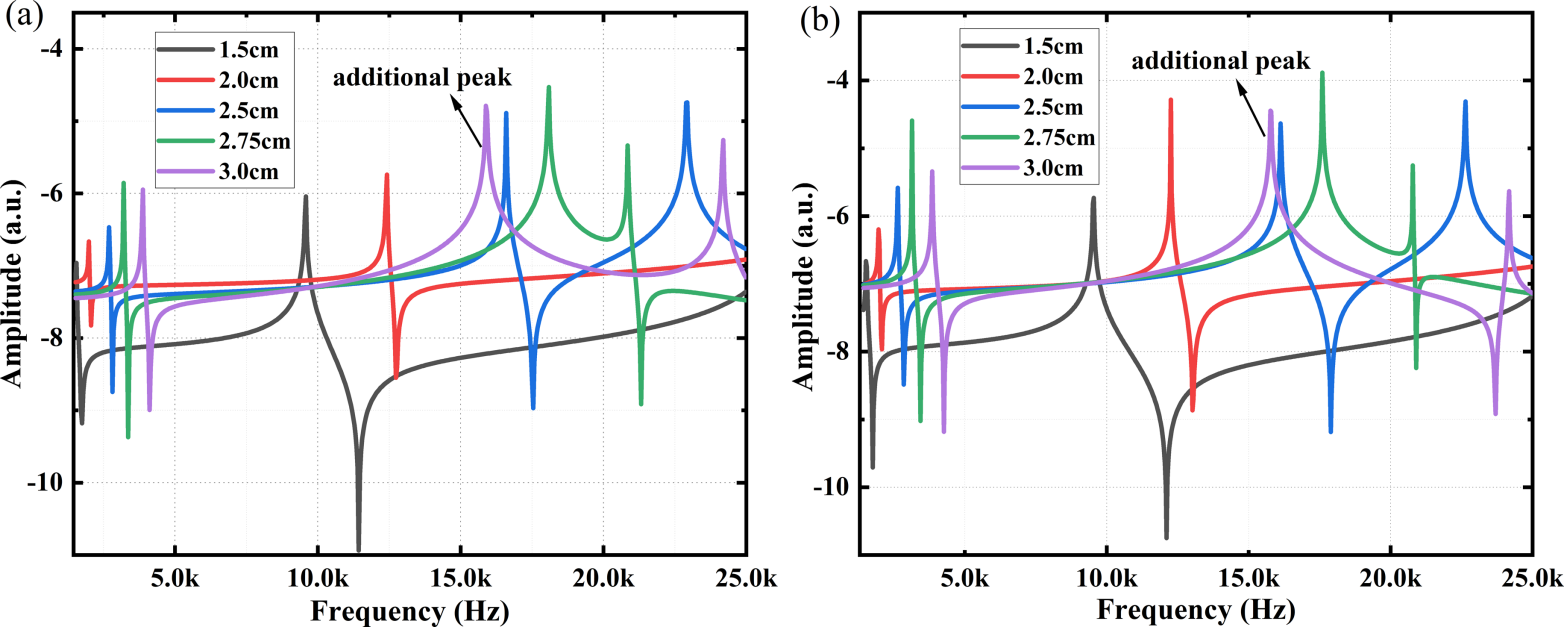
The modal response of the left side of the coupled system for lengths *l* of 1.50, 2.00, 2.50, 2.75, and 3.00 cm. (a) Fixed excitation position and (b) excitation position changing with the bottom support (*l_a_* = 0.5 cm). The additional resonance peak generated at *l* = 3 cm is labeled.

Figure 5 shows the modal response of the left side length *l* for 1.50, 2.00, 2.50, 2.75, and 3.00 cm. When *l* and *L* are not equal, in order to eliminate the influence of the excitation position on the modal response, two scenarios were considered, namely, a fixed excitation position and an excitation position varying with changes in the bottom support device (with *l_a_* = 0.5 cm), as shown in Figure 5a and Figure 5b, respectively. Figure 5 shows that, regardless of the change in excitation position, the modal response is best when *l* is 2.75 cm. The results show that the closer *l* and *L* are, the better the coupling of the system and the stronger the modal response. Figure 5 also shows the resonance peak generated by the additional mode, which shifts to the left with increasing *l*. When *l* is 3 cm, its frequency is already lower than second mode and the response is higher than second mode. This may be due to the fact that the system is not fully coupled; however, the additional resonance peak with good response provides a new option for imaging.

**Figure 6:**
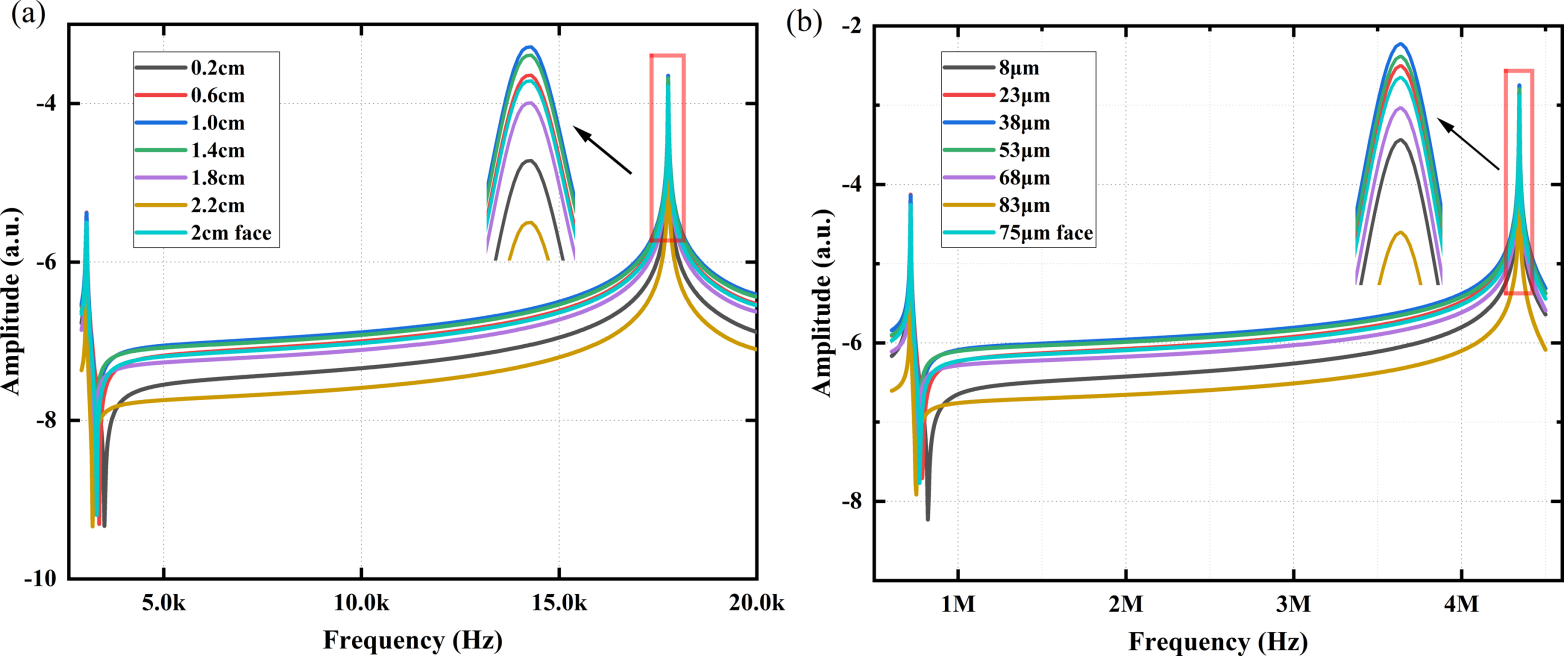
(a) Influence of different excitation positions *l_a_* of the coupled system on the modal response when *l* = 2.75 cm. The “2 cm face” curve represents a 2 cm wide excitation on the left side, and all other curves represent a 0.25 cm wide excitation applied at *l_a_*. The inset shows an enlarged view of the red boxed area. (b) The influence of different excitation positions on the frequency modal response of the microcantilever model.

Figure 6a shows the influence of the excitation position *l_a_* on the modal response when *l* is 2.75 cm. In particular, the 2 cm face curve indicates 2 cm wide excitation to the left side, and all other curves are for 0.25 cm wide excitation. The results show that the largest excitation surface (2 cm face) does not necessarily yield the best modal response. When the excitation position *l_a_* is between 0.6 and 1.4 cm, the modal response is better, especially at around 1 cm, where the frequency modal response is highest. Additionally, we added a simulation of the influence of different excitation positions on the modal response of the microcantilever model (225 × 38 × 7 μm), and the results are shown in Figure 6b. The optimal excitation position is between 23 and 53 μm, and the modal response is highest near 38 μm. This is an approximate match to the scaled-up values of the macroscale cantilevers. This indicates that it is feasible to represent the microcantilever by studying the vibration characteristics of the macroscale cantilever.

## Experimental

### Experimental platform

**Figure 7:**
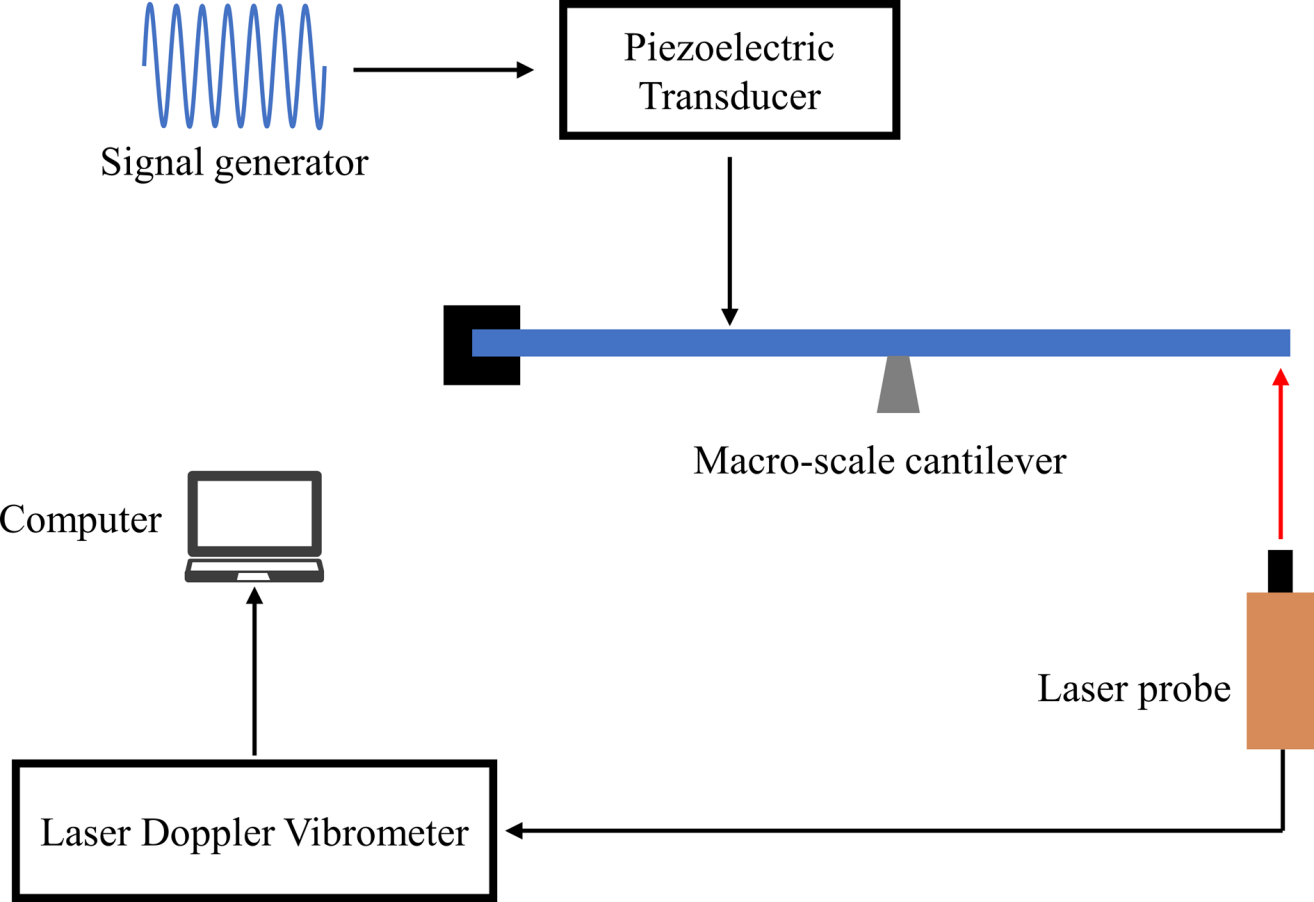
Experimental setup of the macroscale cantilever platform.

**Figure 8:**
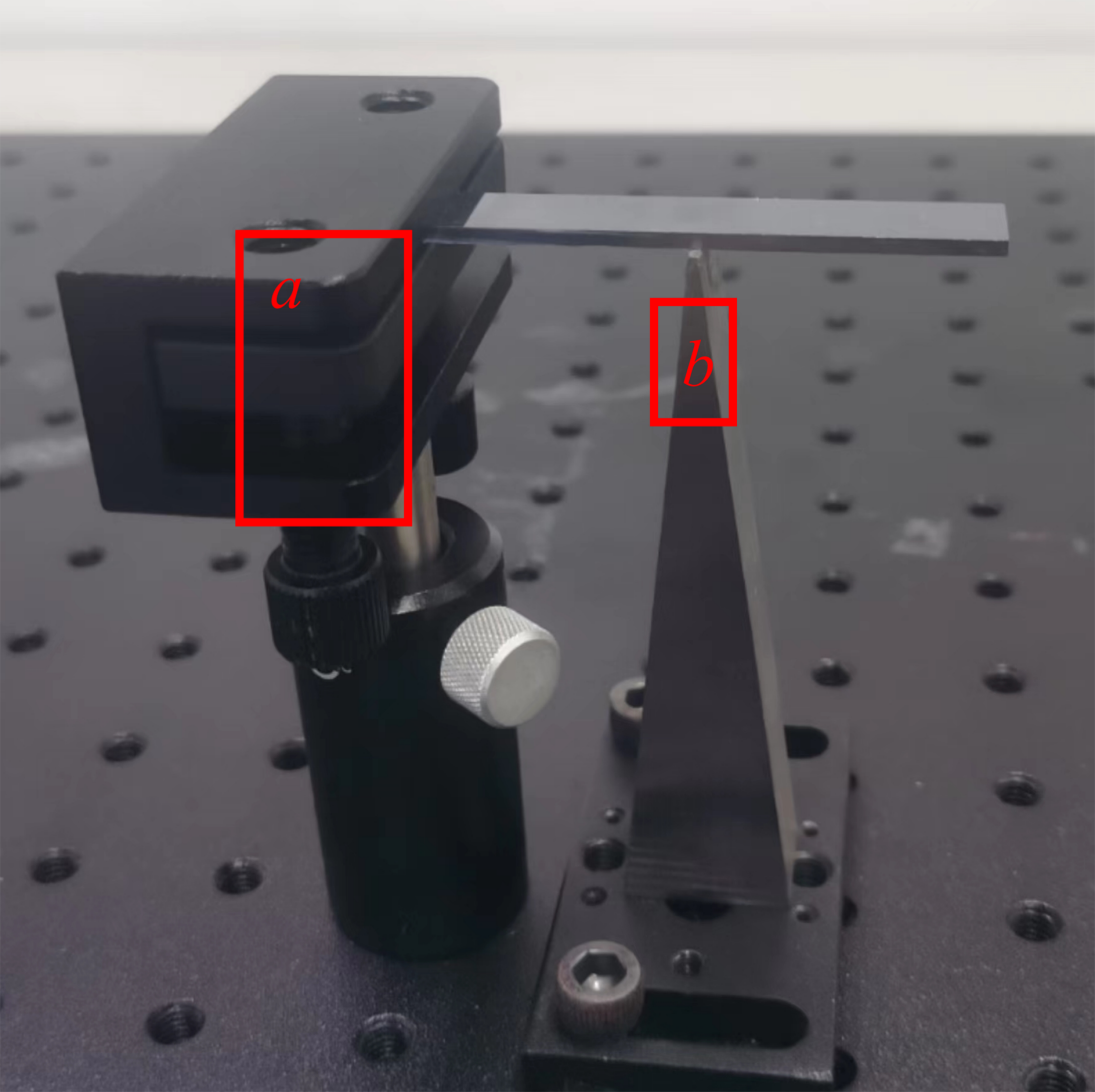
Image of the macroscale cantilever. The left side is clamped by (a) a size-adjustable optical dry plate holder, and the bottom is supported by (b) a machined trapezoidal table with an upper surface area of 10 × 1 mm.

The macroscale cantilever experimental platform setup used to measure the modal response is shown in Figure 7. The experimental setup consists of a macroscale cantilever (6 × 1 × 0.18 cm, silicon content up to 99.9999%) with left-side clamping and a simple bottom support. A photo is shown in Figure 8. In the experiments, the piezoelectric transducer (PZT-5H) was driven by a signal generator (DG1022, Rigol Technologies Co., Ltd.) to apply the excitation force to different positions of the cantilever. 2 cm and 5 cm face excitations were applied with a piezoelectric transducer size of 20 × 14 × 0.2 mm and 50 × 14 × 0.2 mm, respectively. All others excitations were applied with a transducer size of 14 × 2.5 × 0.2 mm with an excitation width of 2.5 mm. The modal response displacement of the free end of the cantilever was measured using a laser Doppler vibrometer (LV-S01, Yuyao Sunny Optical Intelligent Technology Co., Ltd.). The vibrometer has a displacement resolution of 0.008 nm.

### Experimental comparison

**Figure 9:**
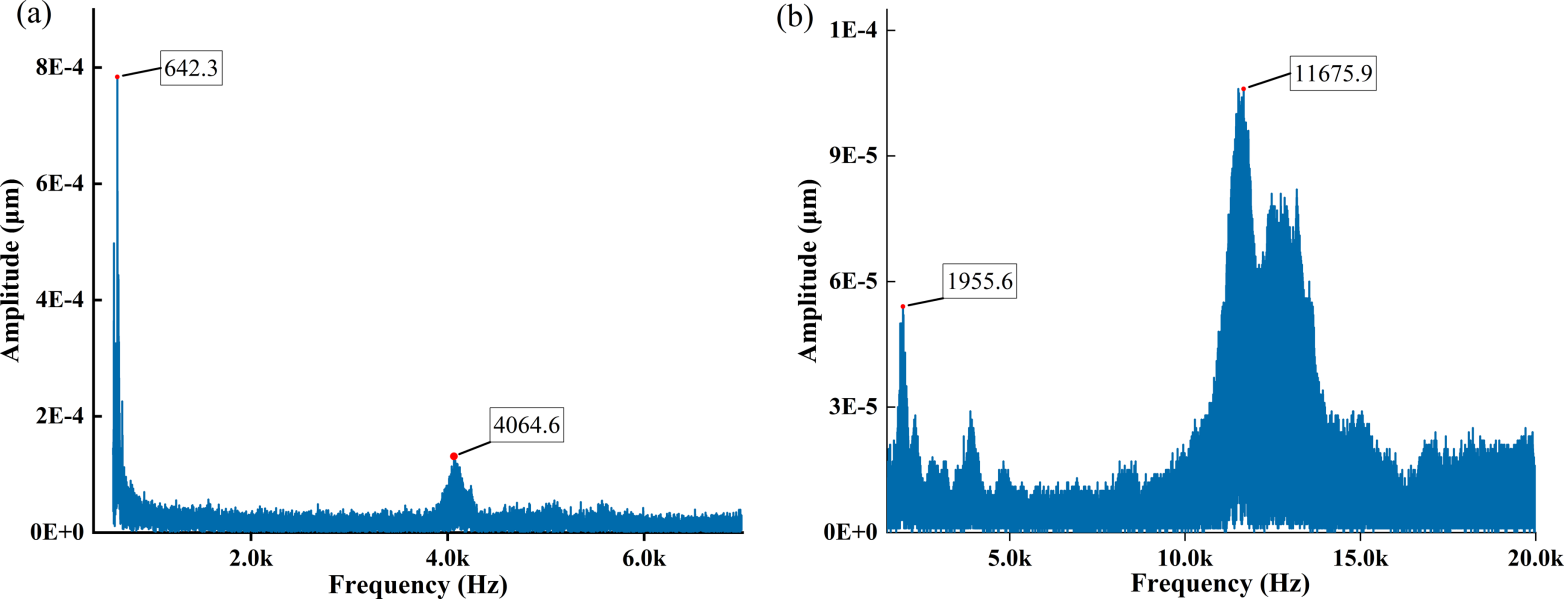
Sweep results for (a) the traditional rectangular cantilever beam and (b) the bridge/cantilever coupled system. The first two orders of modal frequencies are labeled.

First, the amplitude–frequency responses of a traditional cantilever beam and the coupled system can be obtained by the sweep function in the signal generator as shown in Figure 9. The dimensions of the piezoelectric transducers used during the frequency sweep are all 14 × 2.5 × 0.2 mm, which means less mass and less effect on the cantilever modes. The peak-to-peak voltages during the two sweeps are different, and the sweep function of the signal generator is continuously varying. Hence, the amplitude is highly variable and has no reference value.

The first two orders of modal frequencies, *f*_1_ = 642.3 Hz and *f*_2_ = 4064.6 Hz for the traditional cantilever beam and *f*_1_ = 1955.6 Hz and *f*_2_ = 11675.9 Hz for the coupled system, can be obtained from Figure 9. There are some differences in the values of modal frequencies compared to the simulation results, but the modal ratios are approximate. The reasons may be related to the following two aspects: (i) The actual parameters of the cantilever material are different from the simulation parameters. (ii) There is an error in the manual measurement regarding the adjustment of the bottom support device. The latter has a greater effect on the modal frequency.

**Figure 10:**
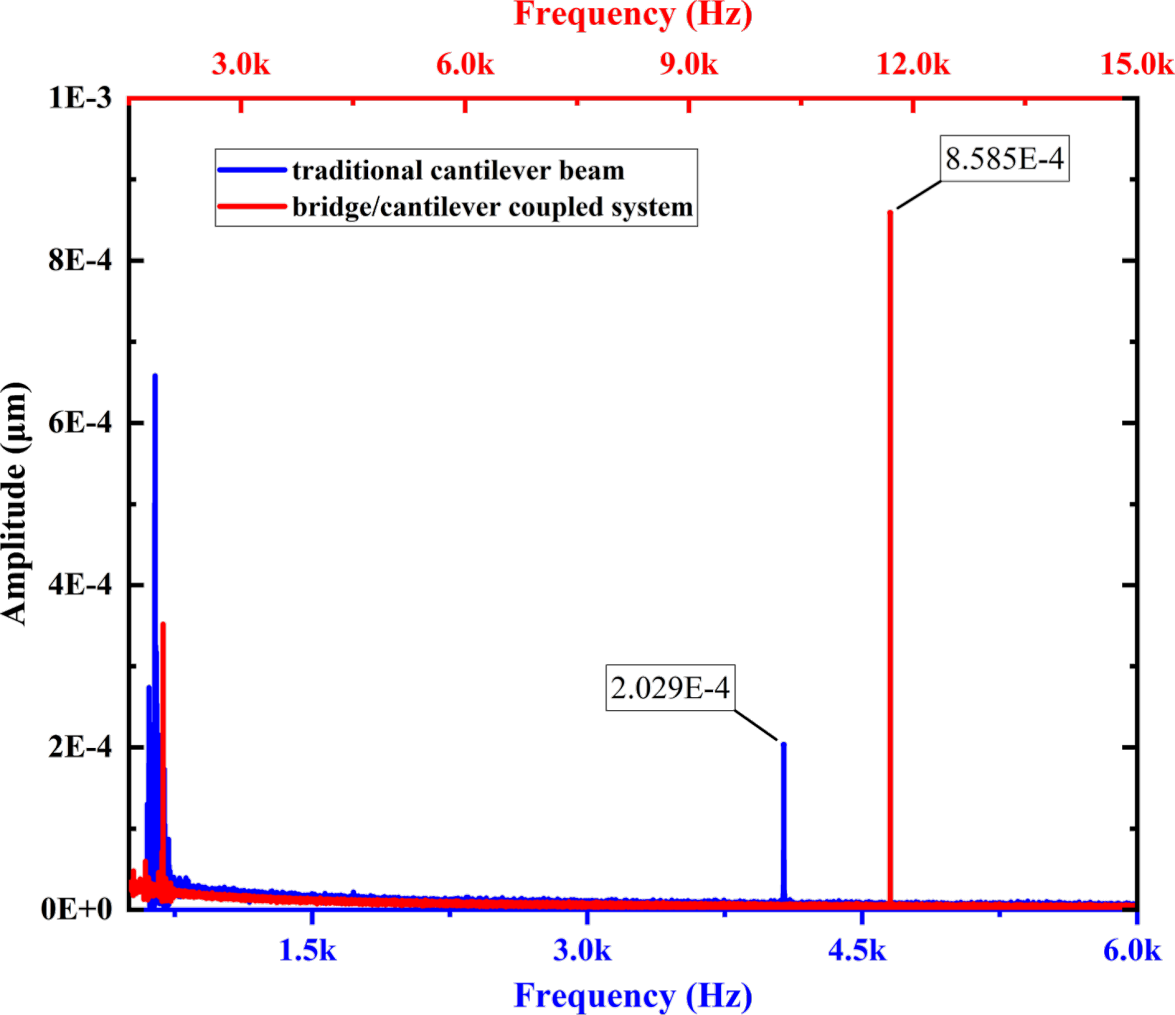
First two orders of frequency response of traditional cantilever beam (blue) and bridge/cantilever coupled system (red). The second-order modal response amplitude values are labeled.

Next, the first two orders of modes of the traditional cantilever beam and coupled system were excited by using piezoelectric transducers (50 × 14 × 0.2 mm and 20 × 14 × 0.2 mm, respectively). The peak-to-peak values of the applied voltages during the measurement of the frequency modal response were all 3 V. The first two orders of the modal responses of the traditional cantilever beam and the coupled system are depicted by blue and red curves, respectively, in Figure 10. We obtained second-order modal response amplitudes of 2.029 × 10^−4^ μm for the traditional cantilever beam and 8.585 × 10^−4^ μm for the coupled system. The modal responsiveness is improved by a factor of 3.23. Compared with the simulation results, the modal responses are basically the same. The fundamental modes are somewhat limited and the higher-order modal response is improved. However, in the experiment, the enhancement factor of the modal response was lower than the simulated values. Mostly, because it is difficult to ensure that the bottom support device is completely at the center position in the experiment, which influences the coupling ability of the coupled system. In addition, it is inevitable that environmental damping has an effect on the results.

### Experimental exploration of optimal conditions

Through preliminary sweeping experiments, we obtained the frequencies of the left-side length *l* at 1.50, 2.00, 2.50, 2.75, and 3.00 cm, as shown in Table 3. In order to minimize the experimental error, a fixed excitation position was used. Compared with the simulation results, the modal frequency changes following the same trend. The modal frequency increases with the increase of *l*. The modal frequencies are not the same, but the difference in their modal ratios is small. Furthermore, from Table 3, it can be seen that a small change in *l* makes a large change in the modal frequency, which is also the reason for the existence of the modal frequency error in this experiment. In addition, we also obtained the additional mode generated at *l* = 3 cm in this sweeping experiment, with a frequency of 10144.0 Hz, lower than the second-mode frequency.

**Table 3:** Experimental results for frequencies and ratios of the first two modes with different lengths on the left side *l*.

*l*/cm	*f*_1_ [Hz]	*f*_2_ [Hz]	modal ratio

1.50	1153.9	7353.1	6.37
2.00	1721.9	10845.3	6.30
2.50	1917.8	11798.2	6.15
2.75	2292.1	13234.1	5.77
3.00	2530.1	16398.6	6.48

**Figure 11:**
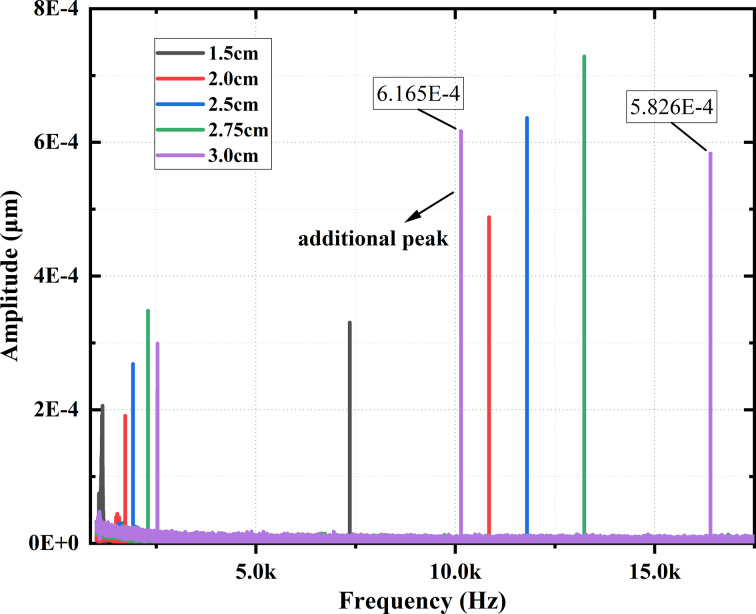
Experimental results of the first two orders of frequency response for different lengths of the left side *l*. The response amplitude of the additional resonance peak and the second mode frequency response amplitude for *l* = 3 cm are labeled.

The frequency response curves obtained in the experiment for different left-side lengths *l* are shown in Figure 11. The results show that the second modal frequency response enhances as *l* increases. When *l* is 2.75 cm, the coupled system has the best coupling and the highest modal response. Moreover, as shown in Figure 11, the response amplitude of the additional resonance peak (labeled on the purple curve) of 6.165 × 10^−4^ μm is larger than the amplitude of the second modal response of 5.826 × 10^−4^ μm when *l* = 3 cm. Therefore, it is feasible to use this resonance peak for imaging, but the imaging effect needs to be tested in actual imaging.

**Figure 12:**
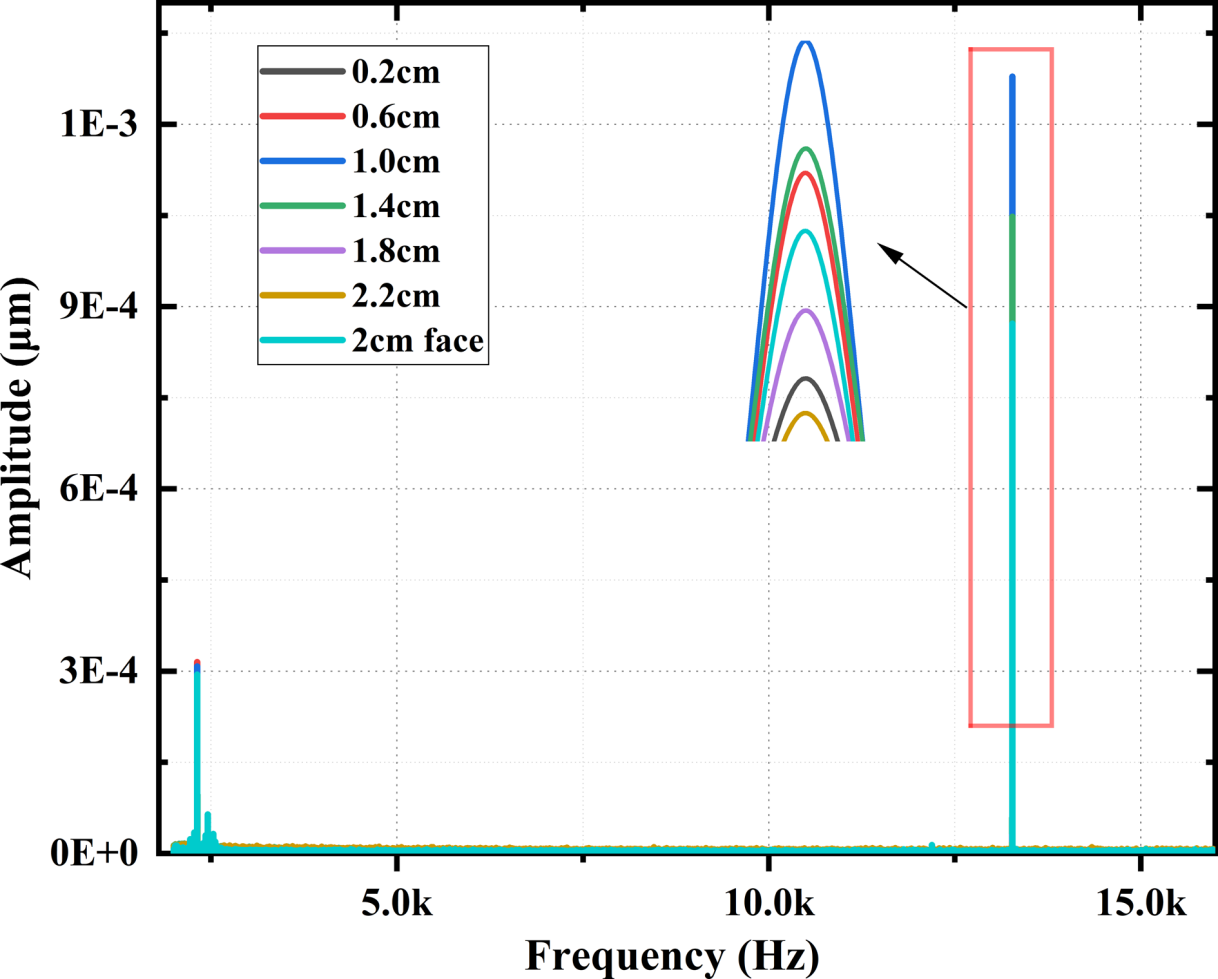
Experimental influence of excitation position *l_a_* on the first two orders of modal response when *l* = 2.75 cm. The inset shows an enlarged view of the red boxed area.

Figure 12 shows the influence of the excitation position *l_a_* on the modal response in the experiment when *l* = 2.75 cm. The size of the piezoelectric transducer used for the 2 cm face excitation is 20 × 14 × 0.2 mm, the size of all others used is 14 × 2.5 × 0.2 mm. The results show that the difference between the modal response at the excitation position *l_a_* at 1.4 cm and at 0.6 cm is smaller than the simulation results. Excitation positions *l_a_* of 0.6–1.4 cm still yield strong modal responses, especially at 1 cm, which is consistent with the simulation results.

## Conclusion

In this paper, we improved the previously proposed model of the bridge/cantilever coupled system and built a macroscale cantilever experimental platform for validation. The model is mainly used to enhance the higher-order modes of the AFM cantilever. In order to approach practical application, the influence of the clamping probe and the size of the excitation surface on the modal response of the cantilever was considered in finite element simulations and experiments. The enhancement of the higher-order modal response capability was quantified by comparing the coupled system model with a traditional cantilever beam. The results of the simulations and experiments show an enhancement of higher order modes by a factor of 7.72 and 3.23, respectively. The modal frequency of the coupled system increases with the length of the left side. When the left-side and the right-side lengths are equal, the modal ratio is minimized, resulting in the best coupling and highest modal response. The excitation position also affects the modal response. Simulation and experimental results show that placing the excitation position of this macroscale cantilever model at 0.6–1.4 cm from the center support device yields better modal response. We also compared it with the microscale coupled model to demonstrate the feasibility of studying the macroscale model to reflect the microscale response. In addition, the coupled system can increase the modal frequency of the original cantilever and potentially generate additional resonance peaks with better response, which will provide new possibilities for high-frequency fast imaging and imaging options. The macroscale experimental study verifies the correctness and shows discrepancies of the coupled system theory and will provide guidance for subsequent practical applications of the microcantilever. Next, we will modify the existing clamping device to integrate it with the support device and design a new excitation method to test the modal response of the coupled system in practical applications of a microcantilever.

## Supporting Information

File 1 Simulations of different cantilevers.

## Data Availability

The data that supports the findings of this study is available from the corresponding author upon reasonable request.
