# Supplementary material for: Enhancing higher-order modal response in multifrequency atomic force microscopy with a coupled cantilever system
Source: Beilstein J Nanotechnol. 2024 Jun 17;15:694–703. doi: 10.3762/bjnano.15.57 (PMC11196946; doi:10.3762/bjnano.15.57)
Supplement: File 1 — Simulations of different cantilevers. [file Beilstein_J_Nanotechnol-15-694-s001.pdf]

BEILSTEIN JOURNAL OF NANOTECHNOLOGY

Supporting Information

for

Enhancing higher-order modal response in multifrequency atomic force microscopy with a coupled cantilever system

Wendong Sun, Jianqiang Qian, Yingzi Li, Yanan Chen, Zhipeng Dou, Rui Lin, Peng Cheng, Xiaodong Gao, Quan Yuan and Yifan Hu

Beilstein J. Nanotechnol. **2024,** *15,* 694–703. doi:10.3762/bjnano.15.57

Simulations of different cantilevers

License and Terms: This is a supporting information file under the terms of the Creative Commons Attribution License (https://creativecommons.org/licenses/by/4.0). Please note that the reuse, redistribution and reproduction in particular requires that the author(s) and source are credited and that individual graphics may be subject to special legal provisions.
The license is subject to the *Beilstein Journal of Nanotechnology* terms and conditions: (https://www.beilstein-journals.org/bjnano/terms)

Table S1: Simulation of the first two orders of modal shapes of microcantilever coupled systems with different cantilever sizes.

**Manufacturer**	**Cantilever type**	**First mode shape**	**Second mode shape**
**Olympus**	AC240FS-B2	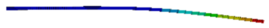	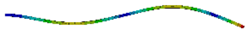
	RC800PS	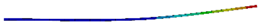	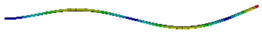
**Asylum**	HQ-75-AU	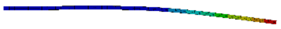	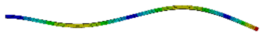
	HQ-150-Au	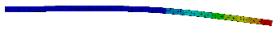	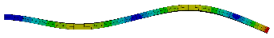
**BudgetSensors**	Tap190-G	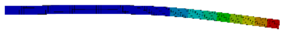	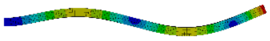
	Tap300-G	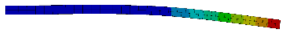	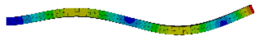
**Bruker**	MPP-21	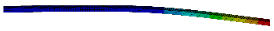	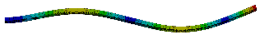
	TESPD	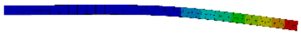	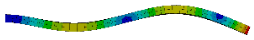
**NanoWorld**	NW-CDT-NCHR	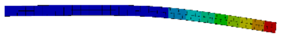	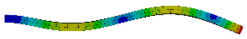
	NW-S-MFMR	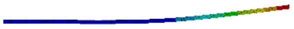	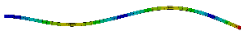
